# Poor knee strength is associated with higher incidence of knee injury in adolescent female football players: The Karolinska football injury cohort

**DOI:** 10.1002/ksa.12567

**Published:** 2024-12-25

**Authors:** Anne Fältström, Martin Asker, Nathan Weiss, Victor Lyberg, Markus Waldén, Martin Hägglund, Ulrika Tranaeus, Eva Skillgate

**Affiliations:** ^1^ Unit of Physiotherapy, Department of Health, Medicine and Caring Sciences Linköping University Linköping Sweden; ^2^ Region Jönköping County Rehabilitation Centre, Ryhov County Hospital Jönköping Sweden; ^3^ Department of Health Promotion Science Musculoskeletal & Sports Injury Epidemiology Center Sophiahemmet University Stockholm Sweden; ^4^ Unit of Intervention and Implementation for Worker Health Institute of Environmental Medicine, Karolinska Institutet Stockholm Sweden; ^5^ Naprapathögskolan Scandinavian College of Naprapathic Manual Medicine Stockholm Sweden; ^6^ Unit of Public Health, Department of Health, Medicine and Caring Sciences Linköping University Linköping Sweden; ^7^ Capio Ortho Center Skåne Malmö Sweden; ^8^ Sport Without Injury Programme (SWIPE) Linköping University Linköping Sweden; ^9^ Department of PNB, SPERIC‐S Swedish School of Sport and Health Sciences Stockholm Sweden

**Keywords:** hip strength, risk factor, screening, soccer

## Abstract

**Purpose:**

To investigate the association between common measures of trunk and lower extremity range of motion (ROM), strength, the results of one‐leg jump tests at baseline and the incidence of subsequent substantial knee injuries in adolescent female football players.

**Methods:**

Players were assessed at baseline regarding (1) ROM of trunk, hip, and ankle; (2) trunk, hip, and knee strength; and (3) one‐leg jump tests. Players were prospectively monitored weekly for 1 year regarding knee injuries and the volume of matches and training. Hazard rate ratios (HRRs) and 95% confidence intervals (CIs) were calculated with Cox regression for the association between the baseline tests and the incidence of substantial knee injury (moderate/severe reduction in training volume or performance, or complete inability to participate in football). Exposures were categorized in tertiles (high, medium and low values). The highest tertile was used as reference.

**Results:**

376 players were included without substantial knee injury at baseline (mean age, 13.9 ± 1.1 years), and 71 (19%) reported at least one substantial knee injury during the follow‐up. Several associations were found; the strongest was that players in the lowest tertile of knee extension strength had a higher incidence of knee injuries than players in the highest tertile (HRR, 2.28; 95% CI, 1.20−4.38). Players in the lowest tertile of trunk rotation ROM in lunge position half‐kneeling (HRR, 0.50; 95% CI, 0.27−0.94) had lower incidence of knee injuries than players in the highest tertile.

**Conclusions:**

Poor knee strength and high trunk ROM were associated with an increased incidence of substantial knee injury in adolescent female football players. Therefore, knee‐strengthening exercises during season may be recommended.

**Level of Evidence:**

Level II.

**Abbreviations**
ACL: anterior cruciate ligament
CI: confidence interval
GHQ‐12: General Health Questionnaire
HR: hazard ratio
KIC: Karolinska Football Injury Cohort
LSI: limb symmetry index
OSTRC‐O: Oslo Sports Trauma Research Centre Overuse
ROM: range of motion
SD: standard deviation

## INTRODUCTION

Football (soccer) is played by more than 13 million female football players in organized clubs globally and more than 3 million players are <18 years old [19]. Adolescent female football players are prone to sustaining injuries, especially to the knee [6, 37]. Injuries often have a multifactorial cause and many factors potentially predispose to injury [33]. Risk factors for injuries in male and female adolescent and adult amateur football players showed that older female players with an existing injury at the start of the season were more prone to new injuries during the season [40]. Investigation of the risk factors for acute knee injuries in adolescent female football players has shown that familial predisposition for anterior cruciate ligament (ACL) injury, the later phase of puberty, and preseason knee complaints increase the risk but with low predictive value [26].

Modifiable risk factors are of particular interest when evaluating preventive interventions. There are now more studies investigating how the trunk and surrounding body areas, such as the hip and ankle, are associated with knee injuries (e.g., ACL injury) [24, 35, 46]. For example, restricted ankle dorsiflexion is more common in those with an ACL injury compared with uninjured controls [46]. Moreover, restricted hip movement, especially restriction of internal and external rotation, showed an association with ACL injury in clinical and radiological studies but was not associated with an increased risk of future ACL injury [35]. There is a complex interaction among various factors that contribute to injuries in adolescent football players [32], and several different screening tests have been utilized to try to identify athletes at higher risk of lower extremity injuries [9]. More knowledge is required regarding whether the results of commonly used screening tests of strength and range of motion (ROM) are risk factors for substantial knee injuries in adolescent female football players. Therefore, the aim of this study was to investigate the association between common screening measures of ROM (in the trunk, hip and ankle), strength measures (for the trunk, hip knee and calf), and one‐leg jump tests at baseline and the incidence of substantial knee injuries regardless of etiology in adolescent female football players. Our hypotheses were that higher strength of the trunk, hip and knee muscles, ROM in adjacent joints, and good performance in one‐leg jump tests would be associated with a lower incidence of any substantial knee injuries.

## METHODS

### Design

This study had a prospective cohort design and was based on the Karolinska Football Injury Cohort (KIC) [44]. The players were included consecutively during 2016−2019 and followed for 1 year after inclusion with weekly online questionnaires.

### Participants

Adolescent female football players from 13 teams were invited, and 418 players aged 12−17 years from 12 teams agreed to participate in the study. For the current study, we excluded players who had pain and/or discomfort in the lower body that hindered them in performing the baseline screening (*n* = 0–10 players in the different tests), did not answer any weekly follow‐up questionnaire (*n* = 18) and/or reported a substantial knee injury (the outcome) (*n* = 24) during the 2 months preceding the baseline.

Oral and written information about the study was given to the players, and signed written consent was obtained from all players and their parents or legal guardians. The study was approved by the Regional Ethical Review Authority in Stockholm (Dnr 2016/1251‐31/4).

### Measurement of exposure and outcome

The procedures for the clinical screening and baseline questionnaires have been described in detail previously in the study protocol [44]. Baseline demographics and information about various football‐related factors were collected with questionnaires at baseline [17, 18, 44] (Table 1). All players were tested indoors during the weekends and at the same time on each day. The players wore indoor shoes, shorts and a t‐shirt. Players completed a standardized 7‐min warm‐up programme comprising 4 min of jogging, 10 squats, 10 squat jumps and 10 unilateral lunges before the testing session. All tests were standardized and divided into nine test stations with one player at each station and one to two test leaders. During the study, undergraduate students of naprapathy were test leaders and were instructed and supervised by three experienced clinicians. Players were assigned randomly to the station they started with the leg that was tested first to reduce the potential influence of a test order effect. The tests took approximately 60 min per player to complete. The test was discontinued if any player experienced pain during the test. Sufficient rest was allowed between the different tests.

**Table 1:** Description of the baseline tests.

Baseline tests	Description
Range of motion (ROM)	
Trunk	Active trunk rotation ROM measured in a sitting and a lunge position half‐kneeling rotation test on a gym mat graded with 5‐degree increments. The player was instructed to maximally rotate alternating between right and left in a seated and lunge position on the dominant and non‐dominant leg, measuring the rotational degrees in the end range. Three repetitions were performed in each direction in the three separate positions. The mean value for the right and left rotation was summarized to obtain the total ROM for each position and was used for the analysis.
Hip: flexion, extension, abduction, internal and external rotation	Passive hip ROM using a universal goniometer was measured in a supine position (flexion and abduction) and in a prone position (extension, internal and external rotation). Three consecutive measurements for each position were performed for both the dominant and the non‐dominant leg and the mean value for each position was used.
Foot: dorsiflexion	The maximal weight‐bearing dorsiflexion ROM was measured in a standing lunge position with a digital inclinometer (Clinometer, Plaincode) in degrees. The player was instructed to lunge forward, keeping her knee aligned with the second toe, until she made contact with the wall, while ensuring that her heel remained on the ground. Three trials were measured and the mean value was used.
Strength	
Back: extensors	Isometric back‐extensor endurance was assessed by the modified Biering‐Sörensen test. The player was instructed to keep her arms folded across the chest throughout the procedure and isometrically maintain the upper body in a horizontal position until failure when the time elapsed was registered.
Trunk: rotational	Isometric trunk rotational strength was measured in a modified standing Wood chopper test utilizing a force gauge to evaluate force output (RS Pro Digital Force Gauge, RS Components Ltd.). The player was instructed to generate force through her trunk and rotate for 5 s, while maintaining straight arms. Three consecutive repetitions were performed in each direction and the maximal force output was used.
Hip: flexion, extension, adduction, abduction	Isometric hip flexion, extension, adduction and abduction strength as well as eccentric hip abduction and adduction strength were measured with a hand‐held dynamometer (MicroFet2, Hoggan Health Industries Inc.). Two submaximal isometric contractions were performed in each direction to familiarize the player with the procedures. Three isometric contractions with gradually increasing power output for 5 s and three maximally eccentric contractions for 3 s were performed in the isometric and eccentric tests, respectively, with a 10‐s rest between contractions. The maximal power output for each position was used.
Knee: extension	Isometric knee extension strength was measured with a hand‐held dynamometer with the player in a seated position with the knee joint at 90° of flexion. Two submaximal isometric contractions were performed in each direction to familiarize the player with the procedures. Three consecutive repetitions were performed and the maximal force output was used.
Calf: heel rises	Ankle plantar flexion muscle endurance was investigated using unilateral weight‐bearing calf heel raises. The number of repetitions accomplished was used.
One‐leg jump tests	
One‐leg long box jump test	The starting position was calculated by dividing the player's height (cm) by 1.6. The test involved standing on one leg in the starting position and then jumping on one leg directed inside the boundaries of the square and maintaining balance after landing. Three warm‐up trials and five consecutive test trials were performed on each leg. The total number of approved trials on each leg was used in the analysis.
Square hop test	The test involved jumping in a clockwise direction on one leg in and out of the square as many times as possible for 15 s. The player performed two warm‐up trials on each foot before executing the test.

The tests included mobility assessment of trunk rotation [2], hip flexion, abduction (in supine position), extension, internal rotation and external rotation (in prone position) [39], and weight‐bearing ankle dorsiflexion [31]. Strength measures included isometric endurance of back extensors (modified Biering‐Sörensen test) [10] and calf heel raises [11]. Isometric trunk rotational strength using a force gauge [1], isometric hip flexion, and extension, isometric and eccentric hip adduction and abduction [43], and isometric knee extension strength were measured with a hand‐held dynamometer (MicroFet2, Hoggan Health Industries Inc.) [29]. The baseline tests are described in Table 1 and in detail in the study protocol [44]. A modified one‐leg long box jump test [34] and a square hop test were also performed [5] to assess the player's unilateral jump performance including agility and neuromuscular control.

Weekly online questionnaires were distributed by e‐mail each Sunday for 52 weeks after baseline. A reminder text message was sent the following Tuesday to non‐responders. The coaches responsible for each team reminded the players to submit these weekly reports. A research assistant also visited each team to collect the remaining reports printed on paper every other week. A Swedish modified version of the Oslo Sports Trauma Research Centre Overuse (OSTRC‐O) injury questionnaire was included in the weekly questionnaire to assess whether players had sustained a football‐related injury [6]. The modified version of the OSTRC‐O included a question regarding absence/reduced participation in training/match due to reasons not related to injuries and the anatomical location of the injuries. In addition, the weekly questionnaires also included questions regarding match and training exposure.

### Definition of injury

The definition of the primary outcome ‘substantial knee injury’ was a football‐related knee injury leading to moderate or severe reduction in training volume, moderate or severe reduction in sports performance, or complete inability to participate in sport [6]. A research assistant contacted players who reported a new substantial knee injury by telephone and conducted a standardized interview with questions about injury localization, type, diagnosis, mechanism, time‐loss, reinjury and medical care. Injury onset was classified as acute (resulting from a specific, identifiable event) or gradual (due to repeated microtrauma without a single, identifiable event responsible for the injury), and both acute and gradual onset injuries were included in the analyses; questions on knee injuries to both dominant (preferred kicking limb) and non‐dominant limbs were also included [21].

### Potential confounders

Potential confounders in the association between the exposures and the outcome were selected a priori with regard to available data, previously published literature and/or biological/theoretical plausibility and with the use of directed acyclic graphs, using the DAGitty software [42]. Identified confounders were age, player position (divided into goalkeepers and outfield players), general health (measured with GHQ‐12 and using a cut‐off score of ≥3) [23], obsessive passion [45], eating habits with regularly skipping a meal during the day [41], impaired sleep [38], onset of menarche or not, and previous time‐loss knee complaints in the past 6 months.

### Statistical methods

Statistical analyses were conducted with SPSS Statistics for Windows (version 27.0; IBM Corp.) and R (version 4.1.2; R Group for Statistical Computing). Means ± standard deviation (SD) were calculated for descriptive statistics. The knee injury incidence was calculated as the total number of players who reported an event of knee injury divided by the total follow‐up time calculated as a sum of hours of training and matches. Cox regression analyses were performed to assess the associations between exposures and outcomes and control for confounding; the results are presented as hazard rate ratios (HRRs) with 95% confidence intervals (CIs). The players' time at risk corresponded to the number of hours of football training and match play from baseline until the first event of knee injury or until the player was censored (dropped out during the study) or until a total time of 52 weeks when the study ended. Players who reported other injuries or other reasons for not participating in football were not censored. All strength measures, except isometric back‐extensor endurance (Biering–Sörensen), were analysed normalized to body weight (N/kg). The ratio between strength in adduction and abduction in the hip was also used in the analysis [48]. The mean values of the dominant and non‐dominant leg in the test results (ROM and strength normalized to body weight) were used in the analysis. For the main analyses, the exposures were categorized in tertiles (high, medium and low values) and the highest tertile was used as reference. The comparisons between the highest tertile and the lowest are presented as forest plots. An additional analysis was performed with standardized mean values of 0 and SD of 1 to facilitate a comparison of results between different measures. Participants with any internal missing data for the exposure variables were excluded from the analyses where data were missing. In the first step, crude (unadjusted) univariable analyses were performed for all variables. In the second step, all identified confounders were included in an adjusted multivariable Cox regression analysis. The proportional hazards assumption was checked by testing for associations between the Schoenfeldt residuals and time, and the assumption was met.

## RESULTS

**Figure 1:**
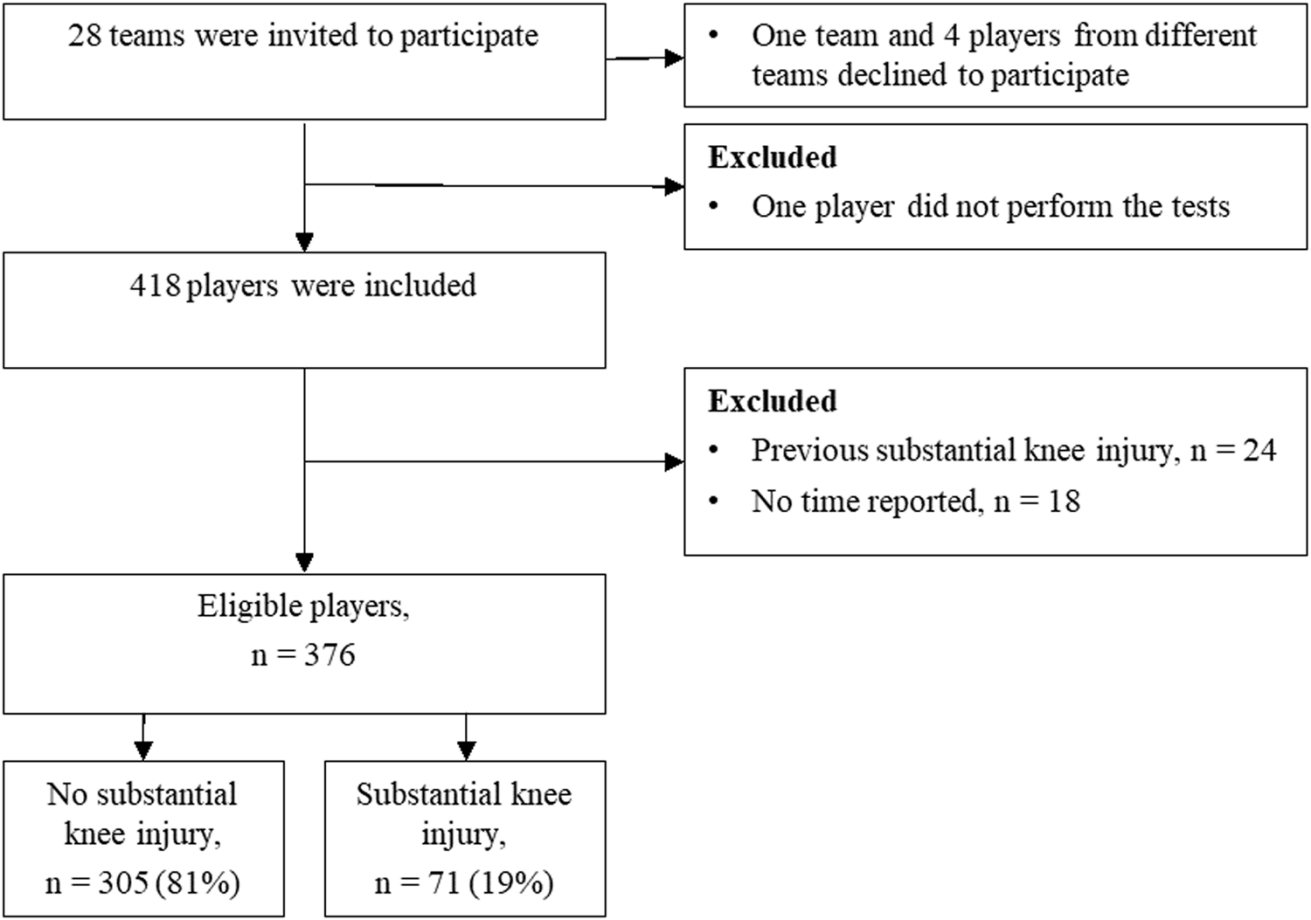
Flowchart of players throughout the study.

Out of 418 players in KIC, 24 were excluded from this study due to substantial knee injury during the 2 months preceding baseline and 18 because no exposure time was reported. Thus, 376 players were eligible and of those, 71 reported a substantial knee injury during the follow‐up period (Figure 1).

Descriptive player data are reported in Table 2, and the results of the tests are presented in Table 3. The mean age of the players was 13.9 ± 1.1 years old and the mean duration of playing in organized football was 7.0 ± 2.1 years. Previous knee complaints leading to reduction in training volume or performance were reported by 9% of the players (Table 2).

**Table 2:** Descriptive characteristics for adolescent female football players at baseline for the total cohort.

	Number	Result
**Age** (years)	376	13.9 ± 1.1
Height (cm)	375	163 ± 6.7
Weight (kg)	375	53 ± 9.0
Body mass index (kg/m^2^)	374	20.0 ± 2.5
**Menarche**, *n* (%)	375	250 (66)
Age at menarche (years)	250	12.5 ± 1.0
Football matches/week	376	1.5 ± 0.6
Football training (h/week)	376	5.0 ± 1.8
Other training with football team (h/week)	375	1.6 ± 1.4
Other training (not football), *n* (%)	375	119 (32)
h/week	117	2.6 ± 2.1
**Playing position**, *n* (%)	372	
Goalkeeper		30 (8)
Defender, midfield, forward		342 (92)
**Eating habits: regularly skipped a meal**, *n* (%)	376	68 (18)
**General health, GHQ‐12 ≥ 3**, *n* (%)	374	89 (24)
**Impaired sleep**, *n* (%)	376	27 (7)
**Passion Scale (0–7) Obsessive**	376	4.8 (1.4)
**Previous knee complaint in the past 6 months**, *n* (%)	375	32 (9)

*Note*: Values are reported as means ± standard deviation or *n* (%). Variables in bold type were adjusted as potential confounders.

Abbreviations: GHQ‐12, General Health Questionnaire‐12.

**Table 3:** Results from the mobility, strength and one‐leg jump tests for adolescent female football players at baseline for the total cohort, players with and without substantial knee injury during follow‐up.

Test	Total number	Total cohort (*n* = 376)	No knee injury (*n* = 305)	Knee injury (*n* = 71)
**Mobility, range of motion**				
Trunk (degrees)				
Seated rotation test	376	141 (21)	140 (20)	144 (24)
In lunge position half‐kneeling rotation test				
Dominant leg in front	375	176 (31)	173 (30)	185 (32)
Non‐dominant leg in front	375	177 (31)	175 (30)	185 (34)
Hip (degrees)				
Flexion	376	121 (9)	121 (9)	121 (9)
Extension	374	23 (9)	23 (9)	23 (8.1)
External rotation	376	45 (10)	46 (10)	44 (11)
Internal rotation	376	45 (8)	45 (8)	44 (8)
Abduction	376	39 (6)	39 (6)	40 (5)
Ankle dorsiflexion (degrees)	364	42 (6)	42 (6)	42 (6)
**Strength tests**				
Trunk, isometric (N/kg)				
Rotational strength	372	1.1 (0.3)	1.1 (0.2)	1.1 (0.3)
Hip, isometric (N/kg)				
Flexion	375	3.3 (0.9)	3.2 (0.9)	3.4 (0.9)
Extension	372	2.5 (0.6)	2.5 (0.6)	2.5 (0.6)
Adduction	373	1.6 (0.6)	1.6 (0.6)	1.6 (0.6)
Adduction, eccentric	373	2.0 (0.7)	2.0 (0.8)	2.0 (0.7)
Abduction	373	1.6 (0.6)	1.6 (0.6)	1.5 (0.6)
Abduction, eccentric	373	1.8 (0.6)	1.8 (0.6)	1.7 (0.7)
Knee (N/kg)				
Knee extension, isometric	374	4.3 (1.1)	4.3 (1.1)	4.0 (0.9)
Endurance				
Isometric back‐extensor endurance (seconds)	376	134 (55)	135 (56)	131 (52)
Calf heel raise test (*n*)	372	12 (12)	13 (13)	11 (5)
One‐leg jump tests (no. of hops)				
One‐leg long box jump (0–5)	367	4 (1)	4 (1)	4 (1)
Square hop test	366	17 (3)	17 (3)	17 (4)

*Note*: Values are reported as means and standard deviation.

In total, 74% of approximately 22,000 weekly reports were answered. The incidence of the first reported substantial knee injury was 1.36/1000 h of training and match play.

Telephone interview information was successfully collected from 55 out of 71 with substantial knee injuries; the remaining 16 injuries were only reported by the online questionnaire which did not include any information about onset, training or match injury or injury mechanism. Twenty‐two of the 55 injuries (40%) were due to acute onset and 33 (60%) had a gradual onset; 22 injuries (45%) occurred during match play and 27 (55%) in training (six missing data); 11 were contact injuries (23%) and 37 (77%) were non‐contact injuries (seven missing data). Two ACL injuries and two meniscus injuries were reported by four players.

**Figure 2:**
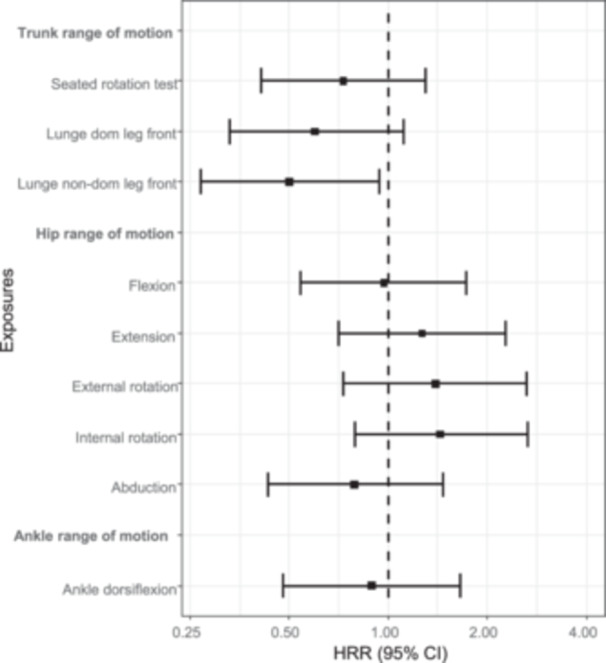
Forest plot of the associations between the results of the range of motion tests and substantial knee injuries (highest tertile [reference]/the lowest). CI, confidence interval; HRR, hazard rate ratio.

**Figure 3:**
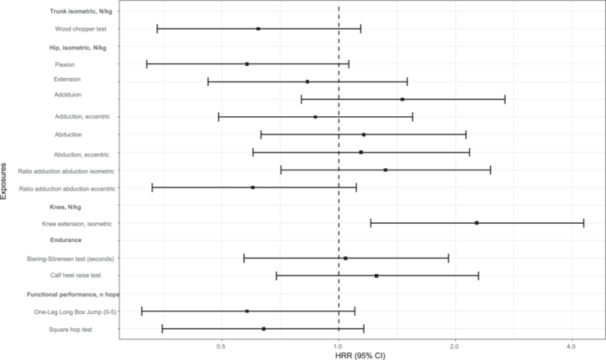
Forest plot of associations between the strength measures and one‐leg jump tests and substantial knee injuries (highest tertile [reference]/the lowest). CI, confidence interval; HRR, hazard rate ratio.

The associations between the ROM in trunk, hip and ankle, strength measures for the trunk, hip, knee and calf, and one‐leg jump tests are presented in Tables 4 and 5 and Figures 2 and 3. Players in the lowest tertile of knee extension strength had a significantly higher incidence of knee injuries than players in the highest tertile (HRR, 2.28; 95% CI, 1.20−4.38). Players in the lowest tertile of rotation ROM in the trunk in lunge position half‐kneeling (HRR, 0.50; 95% CI, 0.27−0.94) had significantly lower incidence of knee injuries than players in the highest tertile. Further, low hip flexion strength, low trunk rotation strength, a low ratio between eccentric strength in adduction and abduction in the hip, and worse results on both one‐leg jump tests seem to be associated with a lower incidence of knee injuries.

**Table 4:** Associations between range of motion and substantial knee injuries.

Range of motion test	Total number	Crude HRR	95% CI (lower–upper)	Adjusted HRR^a^	95% CI (lower–upper)
**Trunk range of motion (degrees)**					
Seated rotation test	376				
High, 150−198°		Reference		Reference	
Medium, 130−149°		0.66	0.37–1.17	0.64	0.35–1.16
Low, 73−129°		0.81	0.47–1.41	0.73	0.41–1.30
In lunge position half‐kneeling rotation test: dominant leg in front	375				
High, 185−278°		Reference		Reference	
Medium, 163−184°		0.77	0.45–1.32	0.60	0.34–1.06
Low, 93−162°		0.64	0.35–1.16	0.60	0.33–1.11
In lunge position half‐kneeling rotation test: non‐dominant leg in front	375				
High, 187−268°		Reference		Reference	
Medium, 162−186°		0.74	0.44–1.26	0.70	0.40–1.22
Low, 93−161°		0.52	0.28–0.96	0.50	0.27–0.94
**Hip range of motion (degrees)**					
Flexion	376				
High, 125−146°		Reference		Reference	
Medium, 118−124°		0.93	0.52–1.65	0.82	0.46–1.47
Low, 94−117°		0.93	0.53–1.63	0.97	0.54–1.72
Extension	374				
High, 28−49°		Reference		Reference	
Medium, 17−27°		1.00	0.56–1.78	1.24	0.68–2.29
Low, 9−16°		1.13	0.65–1.99	1.27	0.71–2.28
External rotation	376				
High, 50−82°		Reference		Reference	
Medium, 40−49°		1.18	0.65–2.12	1.27	0.70–2.31
Low, 22−39°		1.18	0.66–2.12	1.39	0.73–2.63
Internal rotation	376				
High, 48−77°		Reference		Reference	
Medium, 41−47°		1.21	0.67–2.19	1.21	0.66–2.22
Low, 24−40°		1.38	0.77–2.45	1.44	0.79–2.65
Abduction	376				
High, 42−63°		Reference		Reference	
Medium, 37–41°		1.03	0.59–1.80	0.99	0.55–1.77
Low, 22−36°		0.94	0.53–1.68	0.79	0.43–1.47
**Ankle range of motion (degrees)**					
Ankle dorsiflexion	364				
High, 44−62°		Reference		Reference	
Medium, 39−43°		1.22	0.70–2.15	1.21	0.68–2.19
Low, 29−38°		0.94	0.52–1.69	0.89	0.48–1.66

Abbreviations: 95% CI, 95% confidence interval; HRR, hazard rate ratio.

^a^ Adjusted for age, player position (divided into goalkeepers and others), general health (measured with General Health Questionnaire‐12 and using a cut‐off of ≥3), obsessive passion, eating habits with regularly skipping a meal during the day, impaired sleep, onset of menarche or not, and previous knee complaints in the past 6 months) in the Cox regression analyses.

**Table 5:** Associations between strength measures, one‐leg jump tests and substantial knee injuries.

Test	Total number	Crude HRR	95% CI (lower–upper)	Adjusted HRR^a^	95% CI (lower–upper)
**Strength tests**					
**Trunk, isometric**					
Rotational strength (N/kg)	372				
High, 1.2−2.4		Reference		Reference	
Medium, 0.9−1.1		0.86	0.50–1.49	0.72	0.41–1.28
Low, 0.6−0.8		0.68	0.38–1.21	0.62	0.34–1.14
**Hip, isometric**					
Flexion (N/kg)	375				
High, 3.7−5.7		Reference		Reference	
Medium, 2.9−3.6		0.77	0.45–1.33	0.90	0.51–1.59
Low, 0.4−2.8		0.55	0.31–0.99	0.58	0.32–1.06
Extension (N/kg)	372				
High, 2.7−5.0		Reference		Reference	
Medium, 2.2−2.6		0.80	0.45–1.43	0.84	0.46–1.52
Low, 0.4−2.1		0.88	0.50–1.54	0.83	0.46–1.50
Adduction (N/kg)	373				
High, 1.9−3.1		Reference		Reference	
Medium, 1.5−1.8		1.28	0.72–2.30	1.20	0.66–2.21
Low, 0.2−1.4		1.25	0.70–2.26	1.46	0.80–2.68
Adduction, eccentric (N/kg)	373				
High, 2.4−4.6		Reference		Reference	
Medium, 1.8−2.3		0.65	0.36–1.16	0.75	0.41–1.37
Low, 0.3−1.7		0.84	0.49–1.46	0.87	0.49–1.55
Abduction (N/kg)	373				
High, 1.8−3.0		Reference		Reference	
Medium, 1.4−1.7		1.29	0.72–2.32	1.36	0.74–2.49
Low, 0.2−1.3		1.20	0.67–2.17	1.16	0.63–2.13
Abduction, eccentric (N/kg)	373				
High, 2.0−3.7		Reference		Reference	
Medium, 1.7−1.9		1.72	0.96–3.09	1.83	1.00–3.34
Low, 0.2−1.6		1.20	0.65–2.22	1.14	0.60–2.17
Ratio adduction abduction, isometric	374				
High, 1.07−1.45		Reference		Reference	
Medium, 0.95−1.06		1.34	0.76–2.36	1.53	0.84–2.76
Low, 0.67−0.94		1.11	0.62–2.00	1.32	0.71–2.46
Ratio adduction abduction, eccentric	374				
High, 1.23−1.94		Reference		Reference	
Medium, 1.07−1.22		0.81	0.48–1.39	0.71	0.40–1.24
Low, 0.72−1.06		0.54	0.30–0.97	0.60	0.33–1.11
**Knee**					
Knee extension, isometric (N/kg)	374				
High, 4.7−9.3		Reference		Reference	
Medium, 3.9−4.6		1.73	0.93–3.22	1.61	0.85–3.06
Low, 0.5−3.8		1.90	1.03–3.50	2.27	1.21–4.28
**Endurance**					
Isometric back‐extensor endurance (s)	376				
High, 147−414		Reference		Reference	
Medium, 109−146		0.97	0.54–1.76	1.03	0.56–1.89
Low, 29−108		1.18	0.67–2.08	1.04	0.57–1.92
Calf heel raise test (*n*)	372				
High, 13−200		Reference		Reference	
Medium, 8−12		1.35	0.75–2.43	1.41	0.77–2.60
Low, 1−7		1.08	0.60–1.93	1.25	0.69–2.29
**One‐leg jump tests (no. of hops)**					
One‐leg long box jump (0–5)	367				
High, 4.5−5		Reference		Reference	
Medium, 3.5−4		0.66	0.37–1.18	0.67	0.37–1.22
Low, 0−3		0.68	0.37–1.24	0.58	0.31–1.10
Square hop test	366				
High, 19−25		Reference		Reference	
Medium, 16−18		0.63	0.34–1.16	0.63	0.34–1.17
Low, 6−15		0.71	0.40–1.26	0.64	0.35–1.16

Abbreviations: 95% CI, 95% confidence intervals; HRR, hazard rate ratio.

^a^ Adjusted for age, player position (divided into goalkeepers and others), general health (measured with General Health Questionnaire‐12 and using a cut‐off of ≥3), obsessive passion, eating habits with regularly skipping a meal during the day, impaired sleep, onset of menarche or not, and previous knee complaints in the past 6 months) in the Cox regression analyses.

The associations between exposures and outcomes with exposures standardized to have a mean value of 0 and SD of 1 are presented in Supporting Information S1: Table 1).

## DISCUSSION

Lower knee extension strength and higher rotation ROM in the trunk in a lunge position were associated with a higher incidence of substantial knee injuries in adolescent female football players, which partly confirmed our hypotheses. We also found that a lower hip flexion and trunk rotation strength, an imbalance between adduction and abduction eccentric strength in the hip (lower strength in hip adduction than in hip abduction), and worse results in one‐leg jump tests were associated with lower knee injury incidence, but the associations were a bit lower and somewhat more uncertain because the 95% CI included the null association.

### Strength measures

In our study, players with low knee extension strength had more than twice the incidence rate of substantial knee injury. Low knee extension strength has previously been reported to be a risk factor for overuse knee injuries (e.g., patellofemoral pain syndrome and Osgood–Schlatter disease) in early teenage female football players, where a 1 SD increase in quadriceps strength was associated with a 30% decrease risk for overuse knee injuries [37]. Having symmetrical knee extension strength before return to sport after an ACL injury has been reported to decrease the risk of knee reinjury [25]. Therefore, our results strengthen the evidence that specific knee extension strengthening exercises should be included in football training sessions to prevent knee injuries in adolescent female football players [8].

The results were more inconsistent regarding hip strength measures and the incidence of substantial knee injury. Players with a lower hip flexion strength had half the incidence rate of substantial knee injuries with the CI slightly overlapping the HRR one. Our results also indicate that lower eccentric strength in hip adduction than in hip abduction was associated with a lower incidence of knee injury. The mechanisms underlying these eventual associations between high hip flexion strength and knee injuries remain unclear, and further research is warranted to definitively establish this relationship. This is in agreement with findings from several studies focusing on the outcome of ACL injuries that report conflicting results on hip strength as a risk factor for ACL injury [7, 30, 36]. However, the mechanisms of sustaining an ACL injury are different compared with overuse knee injuries. In our study, only two players reported ACL injuries during follow‐up. An association between weakness in the isometric hip external rotator and abductor to patellofemoral pain has been reported previously [13]. Another study reported that an increase in hip flexor and external rotation strength was associated with a decreased risk for overuse injuries in general in young female football players [37]. However, we did not include hip external rotator strength in our study.

### Range of motion

No association was found between ROM measures and the risk of knee injury, except for greater trunk rotational ROM in lunge position half‐kneeling, which was associated with a higher incidence of knee injuries. The seated rotation test was not clearly associated with knee injury. The lunge position half‐kneeling rotation test is a more demanding trunk ROM test, including balance and ROM of the hip and pelvis and could maybe explain the differences regarding the association. However, static measured ROM could not be extrapolated to the dynamic function in explosive actions. In studies evaluating risk factors for ACL injuries, the trunk is often evaluated with 3D motion in activities such as drop vertical jumps, and specific neuromuscular training increased peak trunk flexion and decreased peak trunk extension [27]. Core stability training can modify the biomechanics associated with ACL injury and prevention programmes, including core strength training, might be beneficial for young female athletes [27, 28]. Even patients with patellofemoral pain benefit from hip and core strengthening exercises [14]. However, the association between trunk ROM and core stability is not clear.

We did not find any association between ankle dorsiflexion and knee injuries. Previously, lower ankle dorsiflexion has been reported to be associated with knee dysfunction, such as patella tendinopathy in junior elite basketball players [3], and found to be more common in those with an ACL injury compared with uninjured controls [46]. Previous studies investigating ankle ROM have varying designs, including cohort [3] and case–control [46], with relatively small sample sizes (*n* = 60 and *n* = 70), which make comparisons of results difficult. Decreased ankle dorsiflexion, like decreased hip strength, has been proposed to be associated with greater knee valgus angles and potentially increased risk of ACL injury [46]. Thus, the association with ankle ROM and knee injuries needs to be further evaluated.

### One‐leg jump tests

Lower agility and neuromuscular control (performance) in both the one‐leg jump tests tended to be associated with a lower incidence of knee injury. However, low performance may not be protective for substantial knee injuries; low performance in jump tests could be related to lower match play, that is, low risk for knee injury [15]. The biomechanics and knee joint loading during football are complex, and mechanics and techniques associated with faster cutting performance conflict with reduced knee joint loading (safer cutting mechanics) [12]. The reliability of different functional performance tests and their association with the risk of knee injuries is debatable [15, 16]. Furthermore, ROM and performance in functional tests can change throughout a study because of maturation. In our study, a sub‐cohort was tested after 1 year and generally, fluctuations in ROM were small and probably without clinical importance. However, the results in the one‐leg jump tests improved significantly over 1 year [17] and this should be taken into consideration when interpreting the results.

### Strengths and limitations

Many factors contribute to the risk of sustaining a knee injury [4, 32], and investigating isolated candidate risk factors for sustaining a knee injury in the framework of an injury prevention strategy is probably of limited interest [20]. A strength of this cohort study is the large and representative sample of players in the Stockholm area of Sweden, giving the finding high external validity. Further, 74% of the weekly reports were answered, and only 18 players were excluded, indicating that the risk of selection bias is relatively low. Another strength is the extensive consideration and adjustment of potential confounders for the association between the exposures and the outcome, even though residual and unmeasured confounding may still be present. Other potential confounders considered, but not included in the model, were smoking and alcohol consumption due to the low frequency of consumers and whether ROM affected strength. It was argued that strength tests were performed in neutral positions whereby the ROM would not affect the strength results in the same way as if the tests were performed closer to the limits of participants’ ROM. There is no consensus on whether clinicians should report or measure absolute strength values, strength according to body weight, strength ratios (e.g., hip adductor to abductor, hamstrings to quadriceps), or limb symmetry index (LSI, [dominant/non‐dominant] × 100). We did not report strength ratios or LSI, because LSI can be overestimated due to poor performance of the opposite side [22, 47].

Among the limitations is the risk of non‐differential misclassification of exposure and outcomes. When classifying the ROM measures in tertiles and comparing the highest tertile with the lowest, a potential U‐shaped association may be overlooked. However, comparisons of all three tertiles in Table 4 show that no clear U‐shaped associations were present. In addition, strength and ROM may be misclassified due to the young age of the players. Further, there is a risk of non‐differential misclassification of the outcome when our young players answered the OSTRC‐O questionnaire. The extensive questionnaires used in this study and the frequent follow‐up surveys posed a risk of misclassifying outcomes and confounders, particularly given the young age of the participants. However, because the potential misclassification of exposures and outcomes is probably non‐differential, the risk for bias was considered low. The baseline tests were performed at different time points during the year, and players' strengths may vary over the season. However, the unexposed are compared at the same time point regarding the risk of injury in the longitudinal design. Using the OSTRC‐O questionnaire, the incidence of specific knee injuries could not be established. Detailed information about knee injuries was missing for 16 of the 71 (23%) knee injuries; however, the aim of this study was not to investigate risk factors for specific knee injuries. The data are player‐reported, introducing uncertainty regarding the validity of injury and exposure data. To improve the validity, training and match exposure, and injuries, were reported weekly. The majority of studies examining potential risk factors for knee injuries in female football players have primarily concentrated on ACL injuries. However, ACL injury is a severe and relatively uncommon injury and comprises a part of the overall knee injury burden. Our purpose was to study player‐reported knee complaints leading to moderate, severe reduction in training volume and/or sports performance, or complete inability to participate in sport irrespective of the diagnosis. Different knee injuries and knee complaints may have different aetiologies and injury mechanisms. Various factors could be associated with different knee injuries and must be considered when interpreting the results. Therefore, this study does not inform about the association between the screening tests and the risk of specific knee injuries. Players with substantial knee injury at baseline were not included, but they could have had knee complaints at baseline that were not classified as substantial. Another limitation is that knee ROM, knee flexors and hip external rotation strength were not considered in this study.

## CONCLUSIONS

Poor knee strength and high trunk range of motion were associated with an increased incidence of substantial knee injury in adolescent female football players. Therefore, knee‐strengthening exercises during the season may be recommended.

## AUTHOR CONTRIBUTIONS

All authors planned the study. Eva Skillgate, Nathan Weiss, Victor Lyberg, Martin Asker and Ulrika Tranaeus collected the data. Anne Fältström analysed the data and drafted the manuscript, which was critically revised by all authors. All authors read and approved the final manuscript. Eva Skillgate is the study guarantor.

## CONFLICT OF INTEREST STATEMENT

The authors declare no conflicts of interest.

## ETHICS STATEMENT

The work has been approved by the Regional Ethical Review Authority in Stockholm, Sweden (Dnr 2016/1251‐31/4). The participants were informed about the details of the study. Written informed consent was obtained from all the participants, and the legal guardians also signed if players were <16 years old. Procedures were conducted according to the Declaration of Helsinki. Oral and written information about the study was given to the players, and signed written consent was obtained from all players and their parents or legal guardians.

## Supporting information

Supplementary information.

## Data Availability

Data can be provided on reasonable request.
