# Supplementary material for: Poor knee strength is associated with higher incidence of knee injury in adolescent female football players: The Karolinska football injury cohort
Source: Knee Surg Sports Traumatol Arthrosc. 2024 Dec 25;33(9):3179–93. doi: 10.1002/ksa.12567 (PMC12392389; doi:10.1002/ksa.12567)
Supplement: Supplementary file 1 — Supplementary information. [file KSA-33-3179-s001.docx]

**Supplementary Material**

**Table** (**Supplementary)** Risk hazard rate ratios (HRR) and 95% confidence intervals (CI) between ROM, strength measures and one-leg jump tests respectively, and substantial knee injuries.

| Test | **Total number** | **Crude**  **HRR** | **95% CI**  **(lower–upper)** | **Adjusted HRR** | **95% CI**  **(lower–upper)** |
| --- | --- | --- | --- | --- | --- |
| **Range of motion tests (degrees)** |  |  |  |  |  |
| Trunk |  |  |  |  |  |
| Seated rotation test | 376 | 1.18 | 0.93–1.50 | 1.24 | 0.97–1.59 |
| In lunge position half-kneeling rotation test;  dominant leg in front | 375 | 1.29 | 1.03– .63 | 1.37 | 1.08–1.76 |
| In lunge position half-kneeling rotation test; nondominant leg in front | 375 | 1.26 | 0.99–1.59 | 1.28 | 1.00–1.64 |
|  |  |  |  |  |  |
| Hip range of motion |  |  |  |  |  |
| Flexion | 376 | 1.02 | 0.80–1.28 | 0.98 | 0.76–1.27 |
| Extension | 374 | 0.94 | 0.74–1.18 | 0.86 | 0.68–1.09 |
| External rotation | 376 | 0.93 | 0.73–1.18 | 0.85 | 0.64–1.11 |
| Internal rotation | 376 | 0.91 | 0.72–1.17 | 0.89 | 0.69–1.16 |
| Abduction | 376 | 0.99 | 0.79–1.25 | 1.07 | 0.84–1.37 |
|  |  |  |  |  |  |
| Ankle dorsiflexion | 364 | 1.11 | 0.88–1.40 | 1.06 | 0.83–1.35 |
|  |  |  |  |  |  |
| **Strength tests, N/kg** |  |  |  |  |  |
| Trunk isometric rotational strength | 372 | 1.20 | 0.97–1.48 | 1.24 | 0.99–1.56 |
|  |  |  |  |  |  |
| Hip, isometric |  |  |  |  |  |
| Flexion | 375 | 1.24 | 0.99–1.56 | 1.26 | 0.99–1.59 |
| Extension | 372 | 1.08 | 0.85–1.37 | 1.10 | 0.86–1.41 |
| Adduction | 373 | 0.93 | 0.74–1.18 | 0.89 | 0.70–1.13 |
| Adduction, eccentric | 373 | 1.01 | 0.80–1.28 | 0.97 | 0.77–1.23 |
| Abduction | 374 | 0.94 | 0.74 –1.18 | 0.93 | 0.73–1.17 |
| Abduction, eccentric | 373 | 0.96 | 0.76–1.21 | 0.94 | 0.74–1.19 |
|  |  |  |  |  |  |
| Knee extension, isometric | 374 | 0.79 | 0.63–0.99 | 0.72 | 0.57–0.92 |
|  |  |  |  |  |  |
| **Endurance** |  |  |  |  |  |
| Isometric back-extensor endurance **(seconds**) | 376 | 0.91 | 0.72–1.16 | 0.98 | 0.77–1.25 |
| Calf heel raise test, **n** | 372 | 0.76 | 0.49–1.16 | 0.69 | 0.44–1.11 |
|  |  |  |  |  |  |
| **One-leg jump tests, n hops** |  |  |  |  |  |
| One-Leg Long Box Jump (0-5) | 367 | 1.11 | 0.85–1.45 | 1.24 | 0.93–1.64 |
| Square hop test | 366 | 1.08 | 0.83–1.39 | 1.14 | 0.88–1.49 |

Potential confounders (age, player position (divided into goalkeepers and others), general health (measured with General Health Questionnaire-12 and using a cut-off of ≥3), obsessive passion, eating habits with regularly skipping a meal during the day, impaired sleep, onset of menarche or not, and previous knee complaints in the past 6 months) were included in an adjusted multivariable Cox regression analysis. Abbreviations: CI, 95% confidence intervals; HRR, hazard rate ratio; N, newton; KG, kilogram
